# Mathematical model of a moment-less arch

**DOI:** 10.1098/rspa.2016.0019

**Published:** 2016-06

**Authors:** W. J. Lewis

**Affiliations:** School of Engineering, University of Warwick, Coventry CV4 7AL, UK

**Keywords:** moment-less arch, parabolic form, catenary

## Abstract

This paper presents a mathematical model for predicting the geometrical shapes of rigid, two-pin, moment-less arches of constant cross section. The advancement of this work lies in the inclusion of arch self-weight and the ability to produce moment-less arch forms for any span/rise ratio, and any ratio of uniformly distributed load per unit span, *w*, to uniformly distributed arch weight per unit arch length, *q*. The model is used to derive the shapes of two classical ‘moment-less’ arch forms: parabolic and catenary, prior to demonstrating a general case, not restricted by the unrealistic load assumptions (absence of *q*, in the case of a parabolic form, or no *w*, in the case of a catenary arch). Using the same value of span/rise ratio, and *w*/*q*>1, the behaviour of the moment-less and parabolic arches under permanent loading, (*w*+*q*), is analysed. Results show the former to be developing much lower stresses than its parabolic rival, even when there are relatively small differences in the two geometries; for a medium span/rise ratio of 4 and *w*/*q*=2, differences in the parabolic and moment-less arch geometries would, in practical terms, be viewed as insignificant, but the stresses in them are different.

## 1. Introduction

The question of structural form is viewed mainly as an architectural matter concerned with aesthetics and function. In practice, the shape of an arch is imposed at the conceptual design stage, usually resulting in one of three idealized configurations: circular, parabolic or catenary. These forms become moment-less arches under certain load conditions that can be reproduced in a physical experiment: an inextensible chain that hangs under its own weight produces a catenary shape; a weightless chain carrying a load uniformly distributed along its span gives a parabola, and a uniform load applied normally to a weightless chain produces a circular shape. Inverting these shapes produces moment-less arch forms for the above-described load conditions.

The idea connecting an inverted catenary shape with an optimal arch form dates back to Robert Hooke. As reported by Heyman [1], Hooke demonstrated his idea to the Royal Society in December 1670, but did not published it until 5 years later; it appeared as a record in his diary, and as one of encrypted anagrams in Latin, translated as ‘As hangs the flexible line so, but inverted, will stand the rigid arch’.

Hooke was one of the great experimentalists from whom the idea of using inverted hanging models to shape optimal forms of arches and domes has originated. During the nineteenth and twentieth centuries, significant contributions in this area of experimental form-finding have been made by Gaudi [2,3], Otto [4] and Isler [5,6].

Analytical treatment of the topic was given by Ramsey [7], who derived geometrical configurations of flexible chains and strings that can be used as analogues for optimal arch designs. His derivations included (i) common catenary, (ii) catenary of uniform strength, i.e. a structure made of uniform material, but cross-section area proportional to the level of tension, (iii) elastic catenary—as in case (i) but including elastic elongation of the chain/string. He also demonstrates that for a light string loaded with point loads along its length, the points of load attachment form a shape of a parabolic envelope.

A more recent analytical treatment of arch configuration is due to Osserman [8], who clarifies the confusion over the shape adopted by an architectural landmark: the Gateway Arch in St. Louis. When describing the arch, Osserman replaces a somewhat vague term: ‘loaded catenary’ by a more descriptive and mathematically clear term: ‘flattened catenary’, showing it to be an ideal shape for a free-standing arch of a tapered cross section.

The majority of research related to the optimal form of arches falls into the category of structural optimization. Tyas *et al.* [9] challenge the view that a parabolic funicular is an optimal structural form to carry a uniform load between pinned supports. Using a uniformly distributed load per unit span, *w*, the authors demonstrate that, when the constituent material is equally strong in tension and compression, the optimal (minimal volume) form comprises a central parabolic section and Hencky-net-fan regions near the supports.

Volume minimization tends to be the prime objective of optimization work. Vanderplaats & Han [10] present an optimization technique using a force approximation method iteratively with the finite-element technique to arrive at a minimum volume, but variable cross-section arches that are either pinned, or fixed-ended. However, the structures are subjected to point load and uniformly distributed load per unit span, *w*, only, with arch weight linear density, *q*, yet again, being ignored.

Timoshenko & Young [11] have long advocated the use of moment-less arch forms, so that such structures experience only axial stresses under permanent loads. Axial response to loading is a feature observed in natural objects, and this principle served as motivation for the work presented here. While zero bending moments in the arch may be disputed as the main optimality criterion, in the case of masonry/concrete arches, it is, undoubtedly, a desirable feature.

There is no literature available on the topic of moment-less rib arches that remain moment-less for a given value of the uniformly distributed load per unit span, *w*, relative to self-weight linear density of the arch, *q*, and given span/rise ratio, *l*/*h*. The mathematical model presented in this paper addresses this problem by offering a range of a moment-less arch forms, shaped by the values of these two parameters. The proposed model represents an analytical form-finding approach, in which the structure is shaped by the forces applied to it [12].

Structures under consideration are two-pin, rib arches of constant cross section, made of material that is weak in tension. They are assumed to be sufficiently rigid to prevent deflections under load altering the arch profile significantly. Starting with the equations of equilibrium for a general case of bending, axial force and shear, a necessary and sufficient condition for the case of a pure axial force arch is deduced, which leads to parametrically defined shape equations; equations that require a numerical solution for the chosen parameter.

The proposed moment-less arch model covers three main load cases: *w*/*q*>1, *w*/*q*=1 and *w*/*q*<1. Only the first case is significant in terms of optimal structural design; the other two would produce low stresses in the arch, but might be considered as being of architectural interest. It is shown how the model can be applied to generate two special cases of moment-less arch forms, i.e. parabolic and catenary, before demonstrating the general case involving any *w*/*q* and *l*/*h* ratios. The work is completed by using case studies, which help to assess advantages of moment-less arches over the parabolic forms.

## 2. General equations of equilibrium

**Figure 1.**
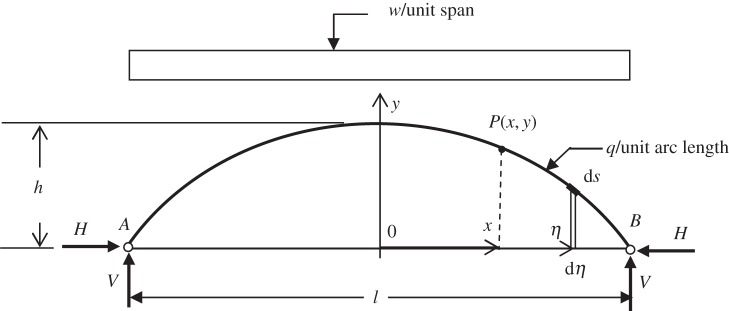
Arch structure.

The arch to be analysed is taken to be a rib arch with self-weight, *q*, taken as a uniform load density per unit arch length. The structure, shown in figure 1, is supporting a deck weight exerting a uniformly distributed load per unit span, *w*.

**Figure 2.**
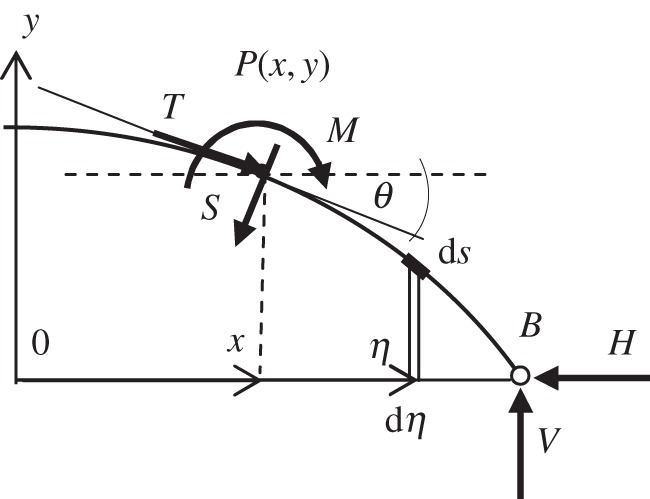
Forces acting on segment *PB*.

Figure 2 illustrates forces that act at on the arch segment, *PB*. These include an axial force *T*, a shear force, *S*, at the face of the section at *P* and a bending moment, *M*. At the pin supports, vertical and horizontal reactions are present, *V* and *H*, respectively. For ease of calculation, variable *η* is introduced to run alongside the *x*-axis. The usefulness of *η* (figures 1 and 2) can be seen with reference to equation (2.4) where it is used to calculate the lever arm for each element of arch length d*s* relative to *P*. The angle *θ* (figure 2) is the acute angle between the tangent at point *P* and the *x*-axis, the overall (un-deformed) arch length is *c*, and *c*(*x*) is the length of arch profile from the apex to an arbitrary point on the arch.

From overall vertical equilibrium of the arch, we have

2.1 $$V=\frac{wl}{2}+q\int_{o}^{c/2}\;ds.$$

For the equilibrium of the segment *PB*, we have

Vertically

2.2 $$-T\sin\theta -S\cos\theta +V-w\int_{x}^{l/2}d\eta -q\int_{c(x)}^{c/2}\;ds=0.$$

Horizontally

2.3 Tcos⁡θ−Ssin⁡θ−H=0.

Rotationally, about *P*

2.4 $$M+Hy-V\left(\frac{l}{2}-x\right)+w\int_{x}^{l/2}\left(\eta -x\right)\;d\eta +q\int_{c(x)}^{c/2}\left(\eta -x\right)ds(\eta )=0.$$

After the substitution for *V* and evaluation of integrals, equation (2.2) becomes

2.2' $$-T\sin\theta -S\cos\theta +wx+q\int_{0}^{c(x)}ds=0.$$

and equation (2.4) becomes

2.4' $$M+Hy-\frac{w}{2}\left\{{\left(\frac{l}{2}\right)}^{2}-x^{2}\right\}-q\left(\frac{l}{2}-x\right)\frac{c}{2}+q\int_{c\left(x\right)}^{c/2}\left(\eta -x\right)\;ds(\eta )=0.$$

Solving for {*T*,*S*,*M*}

2.5 $$\begin{array}{r} \left[\begin{array}{c} T\\ S\\ M \end{array}\right]=\left[\begin{array}{ccc} \cos\theta & \sin\theta & 0\\ -\sin\theta & \cos\theta & 0\\ 0 & 0 & 1 \end{array}\right]\left[\begin{array}{c} H\\ wx+q\int_{0}^{c\left(x\right)\;ds}\\ -Hy+\frac{w}{2}\left\{{\left(\frac{l}{2}\right)}^{2}-x^{2}\right\}+q\left(\frac{l}{2}-x\right)\frac{c}{2}-q\int_{c\left(x\right)}^{c/2}\left(\eta -x\right)\;ds(\eta ) \end{array}\right]. \end{array}$$

Taking *θ* to be the acute angle between the tangent at *P* and the *x*-axis gives

$$\tan\theta =-y^{\prime },$$

and, consequently,

$$\sin\theta =\frac{-y^{\prime }}{{\left(1+y^{\prime 2}\right)}^{1/2}}\;\mathrm{and}\;\cos\theta =\frac{1}{{\left(1+y^{\prime 2}\right)}^{1/2}}.$$

So, explicitly in terms of the curve

2.6 $$\begin{array}{rl} T & =\frac{1}{{\left(1+y^{\prime 2}\right)}^{1/2}}\left[H-y^{\prime }\left(wx+q\int_{0}^{c(x)}ds\right)\right], \end{array}$$

2.7 $$\begin{array}{rl} S & =\frac{1}{{\left(1+y^{\prime 2}\right)}^{1/2}}\left[Hy^{\prime }+wx+q\int_{0}^{c(x)}ds\right] \end{array}$$

2.8 $$\begin{array}{rl} \text{and}\;\;M & =-Hy+\frac{w}{2}\left\{{\left(\frac{l}{2}\right)}^{2}-x^{2}\right\}+q\left(\frac{l}{2}-x\right)\frac{c}{2}-q\int_{c(x)}^{c/2}\left(\eta -x\right)\;ds(\eta ). \end{array}$$

Noting that

$$\int_{c(x)}^{c/2}\left(\eta -x\right)\;ds(\eta )=\int_{x}^{l/2}\left(\eta -x\right){\left(1+y^{\prime 2}\right)}^{1/2}d\eta ,$$

we can differentiate equation (2.8) with respect to *x* to get

2.9 $$M^{\prime }=-Hy^{\prime }-wx-q\int_{0}^{c(x)}ds,$$

and by comparison with equation (2.7), it follows that

$$M^{\prime }=-{\left(1+y^{\prime 2}\right)}^{1/2}S$$

or

$$\frac{dM}{dx}\frac{1}{{\left(1+y^{\prime 2}\right)}^{1/2}}+S=0,$$

and because

2.10 $$\begin{array}{rl} \frac{1}{{\left(1+y^{\prime 2}\right)}^{1/2}} & =\frac{dx}{ds},\\ \frac{dM}{ds}+S & =0. \end{array}$$

If the force at every cross section along the arch is purely compressive, then it is necessary for the shear force *S* to be zero at every cross section. When this is so, then, from equation (2.10), *M* is constant over the length of the arch. Because *M* is necessarily zero at the pin, its value is not only constant, but equal to zero everywhere. Thus, it follows that *a necessary and sufficient condition for a two-pin rib arch to be a pure axial force structure is that the shear force is zero everywhere.* Consequently, we are left only with the expression for *T*

$$T=H\cos\theta +\sin\theta \left[wx+q\int_{0}^{c\left(x\right)}\;ds\right],$$

and, as *S*=0,

$$-H\sin\theta +\cos\theta \left(wx+q\int_{0}^{c\left(x\right)}ds\right)=0,$$

or

$$H=\frac{\cos\theta }{\sin\theta }\left(wx+q\int_{0}^{c\left(x\right)}ds\right),$$

then

2.11 $$T=\frac{1}{\sin\theta }\left(wx+q\int_{0}^{c\left(x\right)}ds\right),$$

and *T* is not zero in general.

It is important to note that this result (equation (2.11)) is quite general, i.e. it applies to any ratio of the uniformly distributed loads, *w*/*q*.

## 3. Classical cases of a moment-less arch

As stated earlier, the two well-known cases of a moment-less arch forms are (i) parabolic, where arch self-weight is assumed to be negligible, and (ii) catenary, where the uniformly distributed deck load is negligible. For completeness, each of these cases is derived below.

### (a) Parabolic arch

Putting *q*=0 in equation (2.7) and taking *S*=0 gives

$$Hy^{\prime }+wx=0$$

or

$$y=-\frac{wx^{2}}{2H}+C,$$

where C is arbitrary constant.

Imposing *y*(*l*/2)=0, leads to

$$C=\frac{w}{2H}{\left(\frac{l}{2}\right)}^{2}$$

and

3.1 $$y=\frac{w}{2H}\left[{\left(\frac{l}{2}\right)}^{2}-x^{2}\right].$$

Equation (3.1) describes the well-known parabolic moment-less arch form. The horizontal reaction, *H*, is not yet known; it is found by imposing the condition that *y*(0)=*h*, giving

3.2 $$H=\frac{wl^{2}}{8h}.$$

### (b) Catenary arch

Putting *w*=0 in equation (2.7) and taking *S*=0 gives

$$Hy^{\prime }+q\int_{0}^{c\left(x\right)}ds=0,$$

or

3.3 $$y^{\prime }=-\frac{q}{H}\int_{0}^{x}{\left(1+y^{\prime 2}\right)}^{1/2}d\eta .$$

On further differentiation

3.4 $$y^{\prime \prime }=-\frac{q}{H}{\left(1+y^{\prime 2}\right)}^{1/2}.$$

Noting that

$$y^{\prime \prime }=\frac{d^{2}y}{dx^{2}}=\frac{d}{dx}\left(\frac{dy}{dx}\right)=\frac{d}{dy}\left(\frac{dy}{dx}\right)\frac{dy}{dx}=\frac{dy^{\prime }}{dy}y^{\prime },$$

and also

$$\frac{d}{dy}y^{\prime 2}=2y^{\prime }\frac{d}{dy}y^{\prime },$$

it follows that

$$y^{\prime \prime }=\frac{1}{2}\frac{d}{dy}y^{\prime 2},$$

or equally

$$y^{\prime \prime }=\frac{1}{2}\frac{d}{dy}\left(1+y^{\prime 2}\right).$$

Using this result and also introducing *β*=*q*/*H* and *p*=1+*y*^′2^ gives

$$\frac{1}{2}\frac{dp}{dy}=-\beta p^{1/2}$$

or

$$\frac{dp}{p^{1/2}}=-2\beta dy,$$

which, after integration gives

3.5 $$p^{1/2}=-\beta y+C,\;C=\text{arbitrary constant}.$$

Imposing *y*(0)=*h*, *y*^′^(0)=0 gives

C=1+βh.

Squaring equation (3.5), substituting for *p*, and separating the variables gives, after integration,

3.6 $$1+\beta \left(h-y\right)=\cos h\beta \left(x+C^{\prime }\right),\;C^{\prime }=\text{arbitrary constant}.$$

Imposing *y*(0)=*h*, gives 1=*cosh* *βC*^′^, and hence *C*^′^=0.

Thus, from equation (3.6), we get

3.7 $$y=\frac{1}{\beta }\left(1+\beta h\right)-\frac{1}{\beta }\cos h\left(\beta x\right),$$

which is a shape of an inverted catenary.

The horizontal reaction *H*, which enters equation (3.7) via *β*, is unknown. As previously, it can be determined by imposing a geometrical condition *y*(±1/2)=0. This gives a transcendental equation for *β*

3.8 $$1+\beta h=\cosh\left(\beta \frac{l}{2}\right),$$

which can be solved numerically.

## 4. General case of a moment-less arch. Derivation of shape equations

This case is the closest to reality, as it includes the self-weight linear density of the arch, *q*, as well as the uniformly distributed deck load, *w*. As will be shown later, this case does not lead to a differential equation that can be integrated in closed form, but it does permit a parametric solution for *y* and *x* separately.

From equation (2.7), putting *S*=0, gives

4.1 $$Hy^{\prime }+wx+q\int_{0}^{x}{\left(1+y^{\prime 2}\right)}^{1/2}d\eta =0,$$

where

$$\int_{0}^{x}{\left(1+y^{\prime 2}\right)}^{1/2}d\eta =\int_{0}^{c\left(x\right)}ds.$$

On differentiating equation (4.1) and putting *α*=*w*/*H*, and *β*=*q*/*H*,

4.2 $$y^{\prime \prime }=-\left[\alpha +\beta {\left(1+y^{\prime 2}\right)}^{1/2}\right],$$

which is the governing nonlinear differential equation for the general case. This equation has a smooth solution that can be rendered unique by choice of appropriate boundary conditions. Considered as a two-point boundary value problem: *y*=0, at *x*=±*l*/2, the graph of the solution, which equation (4.2) indicates is concave down, will have a positive slope for *x*<0, and a negative slope for *x*>0. Because the solution we are seeking is smooth, it follows that *y*^′^=0 at *x*=0, corresponding to maximum rise. In constructing an actual solution, it is found convenient to treat the issue as an initial value problem, with *y*=*h* and *y*^′^=0, at *x*=0, and use the condition at the pin, *y*=0, at *x*=*l*/2, to determine the unknown horizontal reaction.

### (a) Moment-less arch: parametric solution for y

The chosen parameter *z* is such that *z*^2^=1+*y*^′2^, as it can always be chosen to give *z*≥1. It may seem plausible to use *z*=*y*^′^, but this would lead to a singularity at the apex of the arch, where *y*^′^=0.

Recalling

$$y^{\prime \prime }=\frac{dy^{\prime }}{dx}=\frac{1}{2}\frac{d}{dy}\left(1+y^{\prime 2}\right)=\frac{1}{2}\frac{dz^{2}}{dy},$$

equation (4.2) becomes

$$\frac{1}{2}\frac{dz^{2}}{dy}=-\left[\alpha +\beta z\right],$$

which leads to

$$\int \frac{zdz}{\alpha +\beta z}=-\int dy+C,$$

where C is arbitrary constant and

$$\frac{1}{\beta }z-\frac{\alpha }{\beta }\ln\left(\alpha +\beta z\right)=-y+C.$$

Imposing *y*(0)=*h*; *y*^′^(0)=0;

$$C=h+\frac{1}{\beta }-\frac{\alpha }{\beta^{2}}\ln\left(\alpha +\beta \right).$$

Thus, the shape equation for *y* is

$$h-y=\frac{1}{\beta }\left(z-1\right)-\frac{\alpha }{\beta^{2}}\ln\frac{\alpha +\beta z}{\alpha +\beta },$$

or

4.3 $$y=h-\frac{1}{\beta }\left(z-1\right)+\frac{r}{\beta }\ln\frac{r+z}{r+1}.$$

A feature of the solution is that while it depends on *α*/*β*=*r* (and hence the *w*/*q* ratio), it also depends upon *β*=*q*/*H* explicitly.

### (b) Moment-less arch: parametric solution for *x*

The parametric solution for *x* is found following a similar process to that outlined above.

From equation (4.2)

$$\frac{dy^{\prime }}{dx}=-\left[\alpha +\beta {\left(1+y^{\prime 2}\right)}^{1/2}\right],$$

and the separated form is

4.4 $$I_{1}=\int \frac{dy^{\prime }}{\alpha +\beta {\left(1+y^{\prime 2}\right)}^{1/2}}=-\int \;dx+C=-x+C,$$

where *C* is arbitrary constant.

Using *z*^2^=1+*y*^′2^, *y*^′^*dy*^′^=*zdz*, and recalling that *y*^′^<0 and, therefore $y^{\prime }=-\sqrt{z^{2}-1}$, the integral *I*_1_ on the left-hand side of equation (4.4) becomes

$$I_{1}=\int \frac{dy^{\prime }}{\alpha +\beta {\left(1+y^{\prime 2}\right)}^{1/2}}=-\int \frac{zdz}{\sqrt{z^{2}-1}\left(\alpha +\beta z\right)}.$$

With the substitution *z*=*cosh* *θ*

4.5 $$I_{1}=-\int \frac{\cosh\theta d\theta }{\alpha +\beta \cosh\theta }=-\frac{1}{\beta }\int \frac{\alpha +\beta \cosh\theta -\alpha }{\alpha +\beta \cosh\theta }d\theta =-\frac{1}{\beta }\cos h^{-1}z+\frac{2\alpha }{\beta^{2}}\int \frac{d\omega }{\omega^{2}+2r\omega +1},$$

where *ω*=*e*^*θ*^, *r*=*α*/*β*=*w*/*q*

or

4.6 $$\begin{array}{rl} I_{1}= & -\frac{1}{\beta }\cosh^{-1}z+\frac{2\alpha }{\beta^{2}}\int \frac{d\omega }{1-r^{2}+{\left(\omega +r\right)}^{2}}=-\frac{1}{\beta }\cosh^{-1}z+\frac{2\alpha }{\beta^{2}}I_{2}\\ & =-\frac{1}{\beta }\cosh^{-1}z+\frac{2r}{\beta }I_{2}, \end{array}$$

where

$$I_{2}=\int \frac{d\omega }{1-r^{2}+{\left(\omega +r\right)}^{2}},$$

$z=\sqrt{1+y^{\prime 2}}$ and noting that *I*_1_=−*x*+*C*, *C* is arbitrary constant.

The value of the integral *I*_2_ depends upon the sign of 1−*r*^2^. Theoretically, there are three cases to be considered:

(a) *r*>1, i.e. *w*>*q*,
(b) *r*=1, i.e. *w*=*q* and
(c) *r*<1, i.e. *w*<*q*.

Only case (a) is considered to be of structural importance and will be considered here in detail. The remaining two cases may be of architectural interest, but have the unfortunate consequence of the arch carrying the same, or greater, weight than the deck weight. This would lead to under-stressing of the arch cross section/inefficient use of material. Solutions to the last two cases are given in appendix A.

In the case (a): *r*>1, the integral *I*_2_ can be written as

$$\begin{array}{rl} I_{2} & =\int \frac{d\omega }{{\left(\omega +r\right)}^{2}-\left(r^{2}-1\right)}=\frac{1}{2\sqrt{r^{2}-1}}\int \left(\frac{1}{\omega +r-\sqrt{r^{2}-1}}-\frac{1}{\omega +r+\sqrt{r^{2}-1}}\right)\;d\omega \\ & =\frac{1}{2\sqrt{r^{2}-1}}\ln\frac{\omega +r-\sqrt{r^{2}-1}}{\omega +r+\sqrt{r^{2}-1}}=\frac{1}{2\sqrt{r^{2}-1}}\ln\frac{z+\sqrt{z^{2}-1}+r-\sqrt{r^{2}-1}}{z+\sqrt{z^{2}-1}+r+\sqrt{r^{2}-1}}. \end{array}$$

Substituting *I*_2_ into equation (4.6) and noting (4.4) gives

$$-\frac{1}{\beta }\cosh^{-1}z+\frac{r}{\beta {\left(r^{2}-1\right)}^{1/2}}\ln\frac{z+\sqrt{z^{2}-1}+r-\sqrt{r^{2}-1}}{z+\sqrt{z^{2}-1}+r+\sqrt{r^{2}-1}}=-x+C,$$

and using *z*=1 at *x*=0

$$C=\frac{r}{\beta {\left(r^{2}-1\right)}^{1/2}}\ln\frac{\sqrt{r+1}-\sqrt{r-1}}{\sqrt{r+1}+\sqrt{r-1}},$$

the parametric solution for *x* in this case is

4.7 $$x=\frac{1}{\beta }\cosh^{-1}z-\frac{r}{\beta {\left(r^{2}-1\right)}^{1/2}}\ln\left[\frac{\sqrt{r+1}+\sqrt{r-1}}{\sqrt{r+1}-\sqrt{r-1}}\cdot \frac{z+\sqrt{z^{2}-1}+r-\sqrt{r^{2}-1}}{z+\sqrt{z^{2}-1}+r+\sqrt{r^{2}-1}}\right].$$

## 5. Solution process

To find the arch profile, it is necessary to give values of the parameter *z* over the semi-span, in order to generate corresponding values of *x* and *y*. The value of *z* at *x*=0 is 1. The value of *z* at *x*=*l*/2, denoted as $\bar{z}$ is unknown, but can be determined from equations (4.3) and (4.7) as follows. Using equation (4.3) and the boundary condition *y*(*l*/2)=0, we get

5.1 $$h=\frac{1}{\beta }\left(\bar{z}-1\right)-\frac{r}{\beta }\ln\frac{\bar{z}+r}{1+r},$$

and substituting *x*=*l*/2 into equation (4.7) gives

5.2 $$\frac{l}{2}=\frac{1}{\beta }\cos h^{-1}\bar{z}+\frac{1}{\beta }F(\bar{z}),$$

where $F\left(\bar{z}\right)$ is chosen to be the remainder of equation (4.7). In cases of *r*=1, and *r*<1, $F\left(\bar{z}\right)$ is similarly determined using equations (A 1) and (A 2), respectively.

Dividing equations (5.2) by (5.1), we get a single equation for $\bar{z}$

5.3 $$\frac{\rho }{2}=\frac{\cosh^{-1}\bar{z}+F(\bar{z})}{\bar{z}-1-r\ln\frac{\bar{z}+r}{1+r}},$$

where *ρ*=*l*/*h* is the span to height ratio, and $\bar{z}={\left\{1+{\left[y^{\prime }(l/2)\right]}^{2}\right\}}^{1/2}$.

In any iterative solution of equation (5.3), a plausible initial value for $\bar{z}$ is that for the parabolic case, viz., ${\bar{z}}_{\mathrm{initial}}={\left(1+16/\rho^{2}\right)}^{1/2}$. Once $\bar{z}$ is found, *β* is readily obtained from equation (5.1) or (5.2):

5.4 $$\beta =\frac{1}{h}\left[\bar{z}-1-r\ln\frac{\bar{z}+r}{1+r}\right]=\frac{2}{l}\left[\cosh^{-1}\bar{z}+F\left(\bar{z}\right)\right].$$

With *β* known, *H*=*q*/*β* is also known. Using suitable values of the *z* parameter between *z*(0)=1 and $\bar{z}$, the coordinates of the moment-less arch can be found from equations (4.3), and (4.7). In cases: *r*=1 and *r*<1, discussed in appendix A, the relevant equations are (4.3), (A 1) and (A 2), respectively.

## 6. Case studies

The proposed mathematical model is used to produce moment-less arch forms for a range of span/rise ratios, and their shapes are compared with those of parabolic and catenary arches. Detailed stress analysis is carried out for moment-less and parabolic forms, for the ratio of loading *r*=*w*/*q*=2, and span/rise ratio *l*/*h*=2 and 4. The loads have characteristic values, i.e. they are not factored (as they would normally be under design conditions). The arches are of 50 m span, and cross section: 0.680 m deep and 1.470 m wide (giving an area of 1 m^2^). The chosen cross section gives the span/depth ratio of 74, which is within the expected range for an arch structure. Linear weight density of the arch corresponds to that of concrete, with *q*=25 kN m^−1^, in all cases.

**Figure 3.**
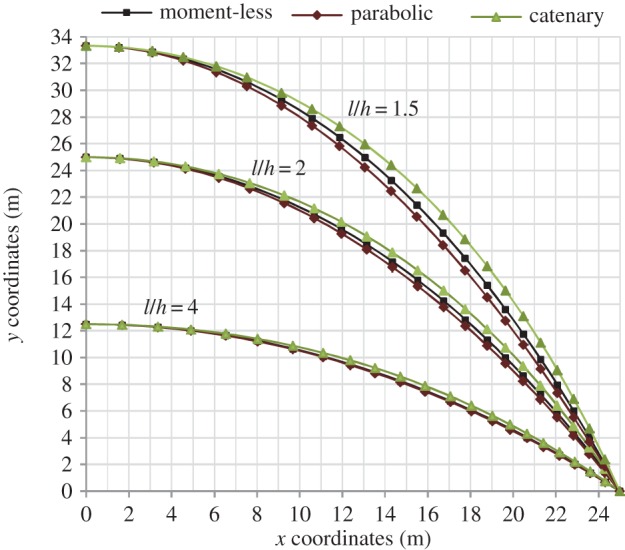
Profiles of moment-less and parabolic arches, respectively, for *r*=2 and varied *l*/*h* ratios. (Online version in colour.)

Figure 3 shows shapes of moment-less, parabolic and catenary arches corresponding to *l*/*h*=1.5, 2 and 4. The shapes were generated using equations (4.7) and (4.3)—in the case of general moment-less forms, and equations (3.1) and (3.2)—in the case parabolic arches. Catenary forms were obtained using equations (3.7) and (3.8). In the case of a general moment-less arch, to ensure a good definition of the shape, non-uniform spacing of the parameter *z* had to be selected, resulting in non-uniform spacing of *x*-coordinates. In addition, the value of the parameter *z* had to be calculated to a high degree of accuracy.

With reference to figure 3, it can be seen that the proposed model predicts correctly the moment-less arch forms to lie between the two limits: catenary (optimal for *w*=0) and parabolic (optimal for *q*=0). At *l*/*h*=2, there is a small, but notable difference between the parabolic and moment-less arch geometries, with the maximum difference in the *y* coordinate of 0.461 m, at *x*=17.768 m (electronic supplementary material, table S1).

Electronic supplementary material, table S1 contains geometrical data for medium rise arches, corresponding to *l*/*h*=4. It is somewhat unexpected to see that for this span/rise ratio, the differences between the parabolic and moment-less arch geometries are, in practical terms, insignificant, with the maximum difference in the *y* coordinate of 0.075 m at *x*=17.062 m.

As parabolic arches would be the usual choice for the case of *r*>1, they are chosen for further stress analysis and comparisons with the moment-less arch forms.

Electronic supplementary material, tables S2 and S2*a* give horizontal and vertical reactions, and axial forces for high rise (*l*/*h*=2), and medium rise (*l*/*h*=4) arches, respectively. Horizontal reactions in the parabolic arch were found using Timoshenko beam theory (linear analysis) offered by GSA software, earlier checked for accuracy using hand calculations. Axial stresses in both types of arches follow axial force values, because their cross section area is 1 m^2^. For the parabolic arch, bending moments are given together with the stresses combining axial force and bending, acting normal to the cross section. Bending moments for the moment-less arch form are not given, as they are zero (within round of error). Reactions for the two types of arches are similar, but it is worth noting that the contribution of arch self-weight to the total vertical reaction, *V* , was found to be significant: around 42% for *l*/*h*=2, and 36% for *l*/*h*=4.

**Figure 4.**
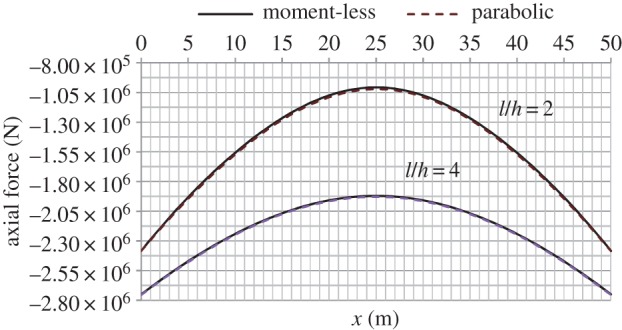
Variation of axial force over the whole arch length, for the two types of arches of high and medium rise, respectively. (Online version in colour.)

**Figure 5.**
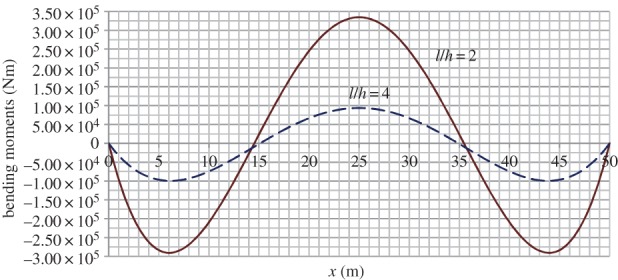
Variation of bending moment in the parabolic arch of high and medium rise, *r*=2. (Online version in colour.)

**Figure 6.**
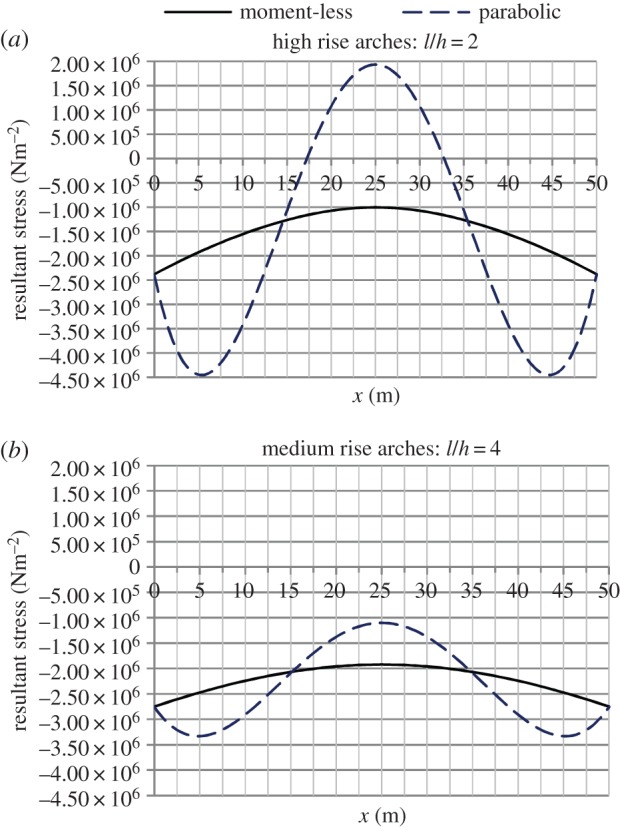
Resultant stresses owing to the axial force in the moment-less arch, and combined axial force plus bending in the parabolic form. (Online version in colour.)

Figures 4–6 provide graphical illustration of data in the electronic supplementary material, tables S2 and S2*a*, but for the whole length of the arch. Results show the axial forces in the moment-less arch to be consistently lower than in the parabolic form, but there is little difference between the two sets of values. At the same time, as can be seen from figure 5, significant bending moments develop in the parabolic arch, within a range of ±300 kNm in the case of *l*/*h*=2, and a third of that value for *l*/*h*=4.

It can be seen that stresses owing to bending combined with the axial force (figure 6 and electronic supplementary material, table S2) produce resultant stresses that vary from tensile to compressive, across the arch cross section. This is in sharp contrast to the moment-less arch behaviour, characterized by pure compression. It is also interesting to note that, for *l*/*h*=4, differences between the parabolic and moment-less arch geometries are, in practical terms, insignificant; yet there are significant differences in stresses developing in the two types of arches, as shown in figure 6.

Results in the electronic supplementary material, tables S2 and S2*a*, show that the largest resultant stresses in the parabolic arch develop in the upper face; it is these stresses that are plotted in figure 6. Comparison of the maximum compressive stresses reveals the parabolic arch to have values at least twice as high as the moment-less arch forms.

The development of tensile stresses impacts the design of arch cross section, as it has to incorporate the presence of steel reinforcement, owing to a low tensile strength of concrete.

## 7. Summary and conclusions

This paper presents a mathematical model for shaping moment-less, two-pin, rib arches of constant cross section. The proposed model includes both arch weight density, *q*, and the uniformly distributed load per unit span, *w*, and its uniqueness lies in the ability to produce moment-less forms for any values of *w*/*q* and span/rise ratio, *l*/*h*. The approach adopted can be compared with an analytical form-finding process in which the shape of a structure is a function of permanent loads applied to it [12]. By eliminating bending moments, the moment-less arch is shaped by the chosen span/rise ratio, and axial force only.

Case studies presented in this paper concern arches made of concrete—a material that is weak in tension. The results show that the arch self-weight makes a significant contribution to the reaction forces; yet, most optimization methods tend to ignore arch self-weight as small.

Compared with the parabolic form, for the case *w*/*q*=2, *l*/*h*=2, the moment-less arch develops much lower compressive stresses and no tension forces under the load considered. In the case of *l*/*h*=4, differences in the parabolic and moment-less arch geometries would, in practical terms, be viewed as insignificant, but the stresses in them are quite different, with the parabolic arch achieving values at least twice as high as the moment-less form.

Further exploration of the effects of *w*/*q* and *l*/*h* on the results will lead to a set of design recommendations, bearing in mind that the resulting moment-less structures must also be safe under defined transient loads appearing during construction, and with live loads acting on part of the span.

It is found that relatively small differences in overall geometries of the two arch forms can result in large differences in stresses. This, combined with the fact that the calculated moment-less arch shapes do not follow simple mathematical functions, raises a question of the accuracy that can be achieved in construction. However, currently, we are equipped with tools that can produce large objects to a ‘mm’ rather than a ‘cm’ accuracy, so it should be possible to meet the construction challenge.

## Supplementary Material

Table 1 Supplementary Info

## Supplementary Material

Table 1a Supplementary Info

## Supplementary Material

Table 2 Supplementary Info

## Supplementary Material

Table 2a Supplementary Info

## Data accessibility

Detailed numerical data regarding arch geometries has been uploaded as part of the electronic supplementary material.

## Appendix A. Solutions for *x*-coordinates for *w*=*q*, and *w*<*q*.

**(a) Case: *w*=*q*; *r*=1**

Here, *I*_2_ (§4b) reduces to

$$I_{2}=\int \frac{d\omega }{{\left(\omega +r\right)}^{2}}=\frac{-1}{\omega +r}=\frac{-1}{z+\sqrt{z^{2}-1}+r},$$

so, inserting *I*_2_ into equation (4.4) and noting that *r*=*α*/*β*=1 gives

$$-\frac{1}{\beta }\cosh^{-1}z-\frac{2}{\beta }\cdot \frac{1}{z+\sqrt{z^{2}-1}+1}=-x+C,$$

and imposing *z*=1 at *x*=0 gives *C*=−2/*β*⋅1/2=−1/*β* and the parametric solution for *x* in this case is

A 1 $$x=\frac{1}{\beta }\cosh^{-1}z+\frac{2}{\beta }\left(\frac{1}{z+\sqrt{z^{2}-1}+1}-\frac{1}{2}\right).$$

**(b) Case: *w*<*q*; *r*<1**

The integral *I*_2_ is in the standard form

$$I_{2}=\int \frac{d\omega }{1-r^{2}+{\left(\omega +r\right)}^{2}}=\frac{1}{\sqrt{1-r^{2}}}{\mathrm{Tan}}^{-1}\left(\frac{\omega +r}{\sqrt{1-r^{2}}}\right).$$

Recalling that *ω*=*e*^*θ*^ and as z=cosh⁡θ, $e^{\theta }=z+\sqrt{z^{2}-1}$, and

$$I_{2}=\frac{1}{\sqrt{1-r^{2}}}{\mathrm{Tan}}^{-1}\left(\frac{z+\sqrt{z^{2}-1}+r}{\sqrt{1-r^{2}}}\right),$$

substituting *I*_2_ into equation (4.4) and noting equation (4.5) gives

$$-\frac{1}{\beta }\cosh^{-1}z+\frac{2r}{\beta }\frac{1}{\sqrt{1-r^{2}}}{\mathrm{Tan}}^{-1}\left(\frac{z+\sqrt{z^{2}-1}+r}{\sqrt{1-r^{2}}}\right)=-x+C.$$

Imposing *z*=1 at *x*=0 and noting that *α*/*β*=*r* gives

$$C=\frac{2r}{\beta }\frac{1}{\sqrt{1-r^{2}}}{\mathrm{Tan}}^{-1}\left(\frac{1+r}{\sqrt{1-r^{2}}}\right)=\frac{2r}{\beta }\frac{1}{\sqrt{1-r^{2}}}{\mathrm{Tan}}^{-1}{\left(\frac{1+r}{1-r}\right)}^{1/2},$$

and the parametric solution for *x* in this case is

A 2 $$x=\frac{1}{\beta }\cosh^{-1}z-\frac{2r}{\beta }\frac{1}{\sqrt{1-r^{2}}}\left[{\mathrm{Tan}}^{-1}\left(\frac{z+\sqrt{z^{2}-1}+r}{\sqrt{1-r^{2}}}\right)-{\mathrm{Tan}}^{-1}{\left(\frac{1+r}{1-r}\right)}^{1/2}\right].$$

## References

[1] Heyman J. 1998 Hooke's cubico-parabolical conoid. *Notes Rec. Royal Soc.* 52, 39–50. (doi:10.1098/rsnr.1998.0033)

[2] Zerbst A 1993 *Antoni Gaudi*, pp. 110–115, English translation: Jones, D. & Gaines, J. Cologne, Germany: Benedikt Taschen Verlag.

[3] Tomlow J 1989 *The model*. Publication IL 34. Stuttgart, Germany: University of Stuttgart.

[4] Otto F, Rasch B 1995 *Finding form. Towards an architecture of the minimal*. Munich, Germany: Deutscher Werkbund Bayern Edition Axel Menges.

[5] Chilton J 2000 *Heinz Isler, the engineer's contribution to contemporary architecture*. London, UK: Thomas Telford, RIBA Publications.

[6] Billington DB 1983 *The tower and the bridge. The new art of structural engineering*. New York, NY: Princeton University Press.

[7] Ramsey AS 1953 *Statics*. Cambridge, UK: Cambridge University Press.

[8] Osserman R. 2010 How gateway arch got its shape. *Nexus Netw.* 12, 167–189. (doi:10.1007/s00004-010-0030-8)

[9] Tyas A, Pichugin AV, Gilbert M 2015 Optimum structure to carry a uniform load between pinned supports: exact analytical solution. *Proc. R. Soc. A* 467, 1101–1120. (doi:10.1098/rspa.2010.0376).

[10] Vanderplaats GN, Han SH 1990 Arch shape optimisation using force approximation methods. *Struct. Optim.* 2, 193–201. (doi:10.1007/BF01748223)

[11] Timoshenko SP, Young DH 1961 *Theory of structures*. New York, NY: McGraw-Hill.

[12] Lewis WJ 2015 Form-finding: an alternative to structural optimisation? *Comput. Technol. Rev.* 11, 121–149. (doi:10.4203/ctr.11.5)

