# Supplementary material for: Mathematical model of a moment-less arch
Source: Proc Math Phys Eng Sci. 2016 Jun;472(2190):20160019. doi: 10.1098/rspa.2016.0019 (PMC4950195; doi:10.1098/rspa.2016.0019)
Supplement: Table 1 Supplementary Info [file rspa20160019supp1.pdf]

Table 1. Detailed arch geometries; *l*/*h* = 2; *r* = 2

	Moment-less		Parabolic	Catenary
z	*x* [m]	*y* [m]	*y* [m]	*y* [m]
1.0000	0.000	25.000	25.000	25.000
1.0068	1.585	24.907	24.900	24.919
1.0273	3.178	24.627	24.596	24.672
1.0580	4.652	24.199	24.134	24.295
1.1024	6.215	23.566	23.455	23.735
1.1536	7.660	22.816	22.653	23.064
1.2218	9.279	21.782	21.556	22.132
1.2901	10.691	20.712	20.428	21.156
1.3583	11.965	19.607	19.273	20.137
1.4265	13.142	18.468	18.092	19.074
1.5016	14.349	17.179	16.765	17.854
1.5801	15.534	15.790	15.348	16.523
1.6654	16.750	14.237	13.778	15.010
1.7405	17.768	12.833	12.372	13.623
1.8190	18.789	11.331	10.879	12.116
1.8872	19.644	9.997	9.564	10.758
1.9555	20.473	8.637	8.234	9.355
2.0237	21.277	7.254	6.891	7.909
2.0920	22.060	5.847	5.534	6.417
2.1602	22.822	4.417	4.166	4.881
2.2285	23.565	2.966	2.787	3.300
2.2967	24.291	1.493	1.398	1.673
2.3649	25.000	0.000	0.000	0.000
