# Supplementary material for: Mathematical model of a moment-less arch
Source: Proc Math Phys Eng Sci. 2016 Jun;472(2190):20160019. doi: 10.1098/rspa.2016.0019 (PMC4950195; doi:10.1098/rspa.2016.0019)
Supplement: Table 1a Supplementary Info [file rspa20160019supp2.pdf]

Table 1a. Detailed arch geometries; *l*/*h* = 4; *r* = 2

	Moment-less		Parabolic	Catenary
z	*x* [m]	*y* [m]	*y* [m]	*y* [m]
1.0000	0.000	12.500	12.500	12.500
1.0022	1.683	12.445	12.443	12.447
1.0086	3.370	12.278	12.273	12.288
1.0183	4.919	12.028	12.016	12.048
1.0323	6.547	11.662	11.643	11.698
1.0485	8.035	11.237	11.209	11.289
1.0701	9.685	10.663	10.624	10.735
1.0916	11.106	10.082	10.033	10.171
1.1132	12.377	9.493	9.436	9.597
1.1347	13.540	8.896	8.833	9.014
1.1584	14.725	8.232	8.163	8.361
1.1832	15.882	7.528	7.455	7.666
1.2102	17.062	6.753	6.678	6.896
1.2339	18.047	6.061	5.986	6.205
1.2587	19.031	5.329	5.256	5.470
1.2802	19.854	4.686	4.616	4.821
1.3018	20.651	4.036	3.971	4.162
1.3233	21.423	3.379	3.321	3.493
1.3449	22.175	2.716	2.666	2.814
1.3664	22.906	2.046	2.006	2.125
1.3880	23.620	1.370	1.342	1.427
1.4095	24.318	0.688	0.673	0.718
