# Supplementary material for: Mathematical model of a moment-less arch
Source: Proc Math Phys Eng Sci. 2016 Jun;472(2190):20160019. doi: 10.1098/rspa.2016.0019 (PMC4950195; doi:10.1098/rspa.2016.0019)
Supplement: Table 2 Supplementary Info [file rspa20160019supp3.pdf]

Table 2. Forces in the moment-less and parabolic arches: *l*/*h* = 2; *r* = 2

Moment-less arch<br>*H* = 1.009E+06 N<br>*V* = 2.160E+06 N		Parabolic arch<br>*H* = 1.025E+06 N<br>*V* = 2.156E+06 N			
x<br>[m]	Axial force<br>[N]	Axial force<br>[N]	Bending moment<br>[Nm]	Resultant stress (upper face)<br>[Nm^{-2}]	Resultant stress (lower face)<br>[Nm^{-2}]
0.000	-1.007E+06	-1.023E+06	3.347E+05	1.931E+06	-3.978E+06
1.585	-1.014E+06	-1.029E+06	3.254E+05	1.843E+06	-3.901E+06
3.178	-1.035E+06	-1.049E+06	2.977E+05	1.579E+06	-3.677E+06
4.652	-1.066E+06	-1.079E+06	2.570E+05	1.189E+06	-3.348E+06
6.215	-1.111E+06	-1.124E+06	2.002E+05	6.430E+05	-2.891E+06
7.660	-1.163E+06	-1.175E+06	1.376E+05	3.929E+04	-2.390E+06
9.279	-1.232E+06	-1.244E+06	5.968E+04	-7.179E+05	-1.772E+06
10.691	-1.301E+06	-1.314E+06	-1.154E+04	-1.416E+06	-1.212E+06
11.965	-1.370E+06	-1.383E+06	-7.553E+04	-2.050E+06	-7.169E+05
13.142	-1.439E+06	-1.452E+06	-1.319E+05	-2.617E+06	-2.889E+05
14.349	-1.515E+06	-1.528E+06	-1.845E+05	-3.157E+06	9.936E+04
15.534	-1.594E+06	-1.608E+06	-2.286E+05	-3.626E+06	4.093E+05
16.750	-1.680E+06	-1.694E+06	-2.634E+05	-4.020E+06	6.306E+05
17.768	-1.755E+06	-1.769E+06	-2.825E+05	-4.264E+06	7.238E+05
18.789	-1.834E+06	-1.848E+06	-2.906E+05	-4.414E+06	7.166E+05
19.644	-1.903E+06	-1.916E+06	-2.877E+05	-4.457E+06	6.225E+05
20.473	-1.972E+06	-1.984E+06	-2.753E+05	-4.415E+06	4.452E+05
21.277	-2.040E+06	-2.052E+06	-2.534E+05	-4.290E+06	1.842E+05
22.060	-2.109E+06	-2.120E+06	-2.220E+05	-4.080E+06	-1.609E+05
22.822	-2.178E+06	-2.187E+06	-1.809E+05	-3.785E+06	-5.907E+05
23.565	-2.246E+06	-2.254E+06	-1.302E+05	-3.404E+06	-1.105E+06
24.291	-2.315E+06	-2.320E+06	-6.992E+04	-2.939E+06	-1.704E+06
25.000	-2.384E+06	-2.386E+06	1.035E-04	-2.387E+06	-2.387E+06

Note: negative stresses imply compression, and positive - tension
