# Supplementary material for: Mathematical model of a moment-less arch
Source: Proc Math Phys Eng Sci. 2016 Jun;472(2190):20160019. doi: 10.1098/rspa.2016.0019 (PMC4950195; doi:10.1098/rspa.2016.0019)
Supplement: Table 2a Supplementary Info [file rspa20160019supp4.pdf]

Table 2a. Forces in the moment-less and parabolic arches: *l*/*h* = 4; *r* = 2

Moment-less arch<br>*H* = 1.920E+06 N<br>*V* = 1.967E+06 N		Parabolic arch<br>*H* = 1.930E+06 N<br>*V* = 1.967E+06 N			
x [m]	Axial force [N]	Axial force [N]	Bending moment [Nm]	Resultant stress (upper face) [Nm^{-2}]	Resultant stress (lower face) [Nm^{-2}]
0.000	-1.921E+06	-1.929E+06	9.385E+04	-1.101E+06	-2.757E+06
1.665	-1.925E+06	-1.933E+06	9.073E+04	-1.132E+06	-2.734E+06
3.334	-1.938E+06	-1.945E+06	8.152E+04	-1.225E+06	-2.665E+06
4.867	-1.956E+06	-1.964E+06	6.808E+04	-1.363E+06	-2.565E+06
6.480	-1.983E+06	-1.991E+06	4.951E+04	-1.554E+06	-2.428E+06
7.957	-2.015E+06	-2.022E+06	2.934E+04	-1.763E+06	-2.281E+06
9.596	-2.056E+06	-2.063E+06	4.598E+03	-2.022E+06	-2.104E+06
11.010	-2.098E+06	-2.105E+06	-1.766E+04	-2.261E+06	-1.949E+06
12.277	-2.139E+06	-2.146E+06	-3.738E+04	-2.476E+06	-1.816E+06
13.438	-2.181E+06	-2.188E+06	-5.449E+04	-2.669E+06	-1.707E+06
14.623	-2.226E+06	-2.233E+06	-7.023E+04	-2.853E+06	-1.613E+06
15.782	-2.274E+06	-2.281E+06	-8.315E+04	-3.015E+06	-1.547E+06
16.967	-2.326E+06	-2.332E+06	-9.302E+04	-3.153E+06	-1.511E+06
17.957	-2.371E+06	-2.378E+06	-9.806E+04	-3.244E+06	-1.512E+06
18.949	-2.419E+06	-2.425E+06	-9.959E+04	-3.304E+06	-1.546E+06
19.779	-2.460E+06	-2.467E+06	-9.778E+04	-3.330E+06	-1.604E+06
20.584	-2.502E+06	-2.508E+06	-9.300E+04	-3.329E+06	-1.687E+06
21.365	-2.543E+06	-2.549E+06	-8.522E+04	-3.301E+06	-1.797E+06
22.127	-2.585E+06	-2.590E+06	-7.439E+04	-3.247E+06	-1.933E+06
22.869	-2.626E+06	-2.632E+06	-6.048E+04	-3.166E+06	-2.098E+06
23.595	-2.667E+06	-2.673E+06	-4.347E+04	-3.057E+06	-2.289E+06
24.304	-2.709E+06	-2.714E+06	-2.332E+04	-2.920E+06	-2.508E+06
25.000	-2.750E+06	-2.755E+06	1.049E-01	-2.755E+06	-2.755E+06

Note: negative stresses imply compression, and positive - tension
